# Imperforate Anus Associated with Eventration of Diaphragm

**Published:** 2013-01-01

**Authors:** Bilal Mirza

**Affiliations:** Department of Pediatric Surgery, The Children’s Hospital and the Institute of Child Health Lahore, Pakistan

**Dear Sir**

Anorectal malformations (ARM) are associated with a myriad of congenital anomalies. The prevalence rates of associated anomalies range from 20% to 80%, commonest being urogenital anomalies. Other important associations and syndromes include Down syndrome and VACTERL (vertebral, anorectal, cardiac, trachea-esophageal, renal, limb) anomalies [1]. ARM has rarely been described in association with diaphragmatic anomalies [2-7]. Fewer than half a dozen cases of diaphragmatic hernia are recorded in the literature; but none of diaphragmatic eventration.

A 36-hour-old male neonate presented with imperforate anus and abdominal distension. There was no meconuria. Antenatal fetal anomaly scanning had not been done. Except for mild tachypnea (respiratory rate 60/min) physical examination of chest was nothing abnormal. Ultrasound of the abdomen did not reveal any anomaly. Invertogram (Fig. 1) showed a high variety imperforate anus. A sigmoid colostomy was performed. The colostomy started moving and patient was allowed orally the following day. The patient developed respiratory distress after starting feeds. A chest radiograph showed eventration of left hemidiaphragm (Fig. 2). A nasogastric tube inserted for gastric decompression alleviated the respiratory distress. The patient underwent plication of the diaphragm electively on 8th day of life. Postoperative recovery was uneventful. The patient is lost to follow-up.

**Figure:**
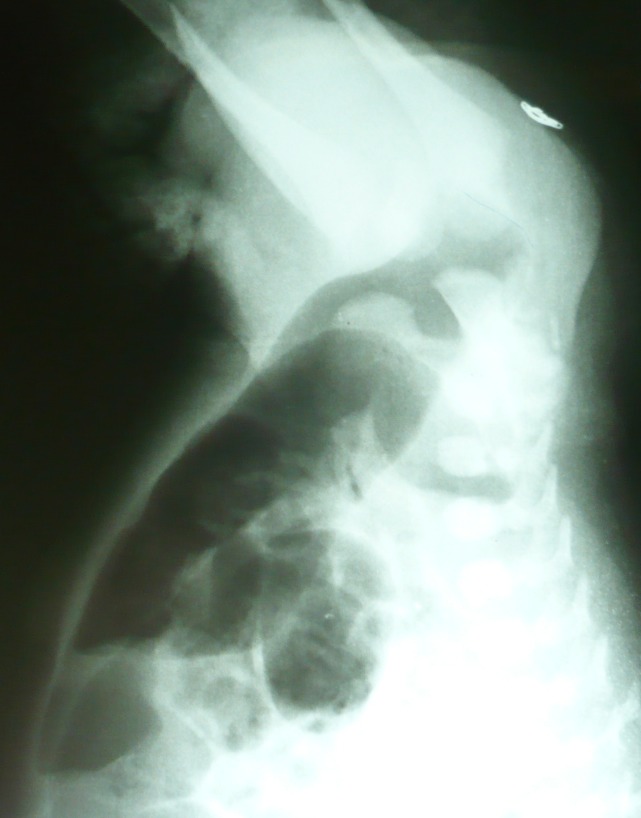
Figure 1: High variety Imperforate anus.

**Figure:**
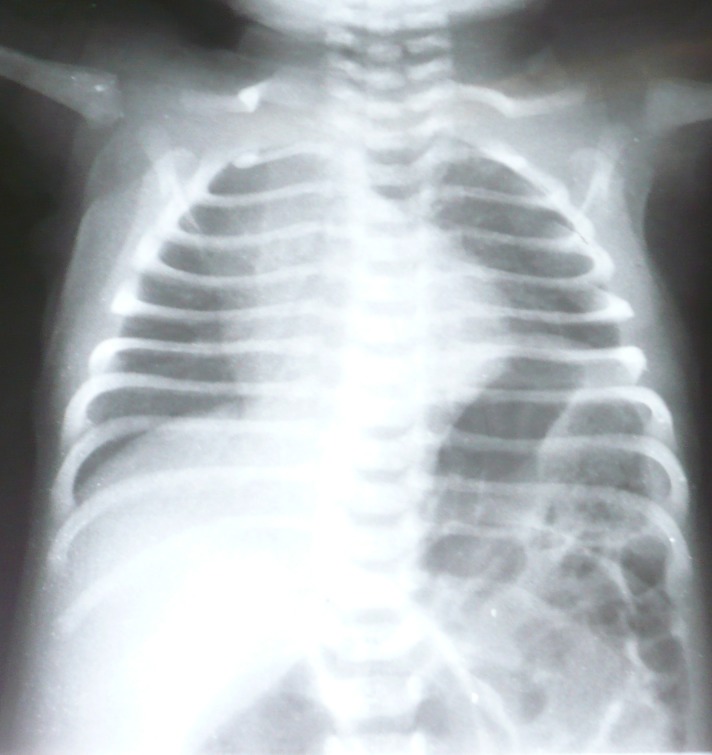
Figure 2: Elevated left hemidiaphragm in the same patient.

Eventration of diaphragm usually presents with respiratory distress and recurrent respiratory tract infections although it may remain asymptomatic when it is diagnosed incidentally on radiological investigations. In the index case, respiratory distress developed after initiating feeds led us to investigate the cause. Eventration of diaphragm is seldom reported with imperforate anus. Ein SH [3] has reported the only case of imperforate anus associated with multiple anomalies including rectal and colonic atresias, syndactyly, and eventration of right hemidiaphragm. A literature search did not retrieve any case of imperforate anus associated with eventration of left hemi diaphragm.

## Footnotes

**Source of Support:** Nil

**Conflict of Interest:** The author belongs to the editorial team; however, the manuscript is independently dealt by other editors and he is not involved in decision making of the manuscript.
